# Clinical implementation of standardized neurocognitive assessment before and after radiation to the brain

**DOI:** 10.1016/j.ctro.2023.100664

**Published:** 2023-07-22

**Authors:** C.M.L. Zegers, C. Offermann, J. Dijkstra, I. Compter, F.J.P. Hoebers, D. de Ruysscher, M.M. Anten, M.P.G. Broen, A.A. Postma, A. Hoeben, K.E. Hovinga, W. Van Elmpt, D.B.P. Eekers

**Affiliations:** a Department of Radiation Oncology (Maastro), Maastricht University Medical Center+, GROW-School for Oncology and Reproduction, Maastricht, the Netherlands; b Department of Medical Psychology Maastricht University Medical Center+, MHeNs School for Mental Health and Neuroscience, Maastricht, the Netherlands; c Department of Neurology, GROW – School for Oncology and Reproduction, Maastricht University Medical Center+, Maastricht, Netherlands; d Department of Radiology & Nuclear Medicine, Maastricht University Medical Center+, MHeNs School for Mental Health and Neuroscience, Maastricht, the Netherlands; e Dept of Medical Oncology, GROW-School for Oncology and Reproduction, Maastricht University Medical Center+, Maastricht, the Netherlands; f Department of Neurosurgery, Maastricht University Medical Center+, P.O. Box 5800, 6202 AZ Maastricht, the Netherlands

**Keywords:** Radiotherapy, Brain, Cognition, Memory, Attention, Implementation

## Abstract

**Background:**

Radiotherapy induced impairment of cognitive function can lead to a reduced quality of life. The aim of this study was to describe the implementation and compliance of standardized neurocognitive assessment. In addition, the first results of cognitive changes for patients receiving a radiation dose to the brain are described.

**Materials and methods:**

Patients that received radiation dose to the brain (neuro, head and neck and prophylactic cranial irradiation between April-2019 and Dec-2021 were included. Three neuro cognitive tests were performed a verbal learning and memory test, the Hopkins Verbal Learning Test; a verbal fluency test, the Controlled Oral Word Association Test and a speed and cognitive flexibility test, the Trail Making Test A&B. Tests were performed before the start of radiation, 6 months (6 m) and 1 year (1y) after irradiation. The Reliable Change Index (RCI) between baseline and follow-up was calculated using reference data from literature.

**Results:**

644 patients performed the neurocognitive tests at baseline, 346 at 6 months and 205 at 1y after RT, with compliance rates of 90.4%, 85.6%, and 75.3%, respectively. Reasons for non-compliance were: 1. Patient did not attend appointment (49%), 2. Patient was unable to perform the test due to illness (12%), 3. Patient refused the test (8 %), 4. Various causes, (31%). A semi-automated analysis was developed to evaluate the test results. In total, 26% of patients showed a significant decline in at least one of variables at 1y and 11% on at least 2 variables at 1y. However, an increase in cognitive performance was observed in 49% (≥1 variable) and 22% (≥2 variables).

**Conclusion:**

Standardized neurocognitive testing within the radiotherapy clinic was successfully implemented, with a high patient compliance. A semi-automatic method to evaluate cognitive changes after treatment was defined. Data collection is ongoing, long term follow-up (up to 5 years after treatment) and dose–effect analysis will be performed.

## Highlights

• Successful implementation of standardized neurocognitive testing within the radiotherapy clinic.
• High patient compliance >75% after 1 year.
• 1 year after treatment significant changes in cognitive performance is observed.
• Data collection is ongoing, long-term follow-up will be performed.

## Introduction

For the majority of brain and head and neck tumors, radiotherapy is part of the standard treatment. Although the radiation treatment is focused on the tumor, it inevitably leads to irradiation of healthy brain tissue, which can cause radiation induced brain-injury. One of the potential side effects of radiation to the brain is the impairment in cognitive functioning. Memory, learning, processing speed, executive function and attention can be affected cognitive domains after radiotherapy to the brain [1], [2]. Cognitive deficits can occur in up to 50–90% of patients that survive >6 months after treatment with high dose radiotherapy for primary or metastatic brain tumors. [3]. Patients with head and neck cancer can also receive a significant radiation dose to brain tissue, and are therefore, in combination with the use of chemotherapy at risk for adverse brain affects like impaired cognitive functioning [4], [5]. A reduction in cognitive function can lead to a premature loss of independence and reduced quality of life.

The extent of these delayed adverse effects can vary with the patients age, education level, and baseline neurocognitive performance as well as the location of the tumor and the radiation dose to the hippocampus [6]. Understanding the mechanism underlying radiation induced cognitive decline is complex and which brain areas are important to spare are subject of research [7], [8]. Important aspect is the definition of organs-at-risk within the brain. The European Particle Therapy network (EPTN) international neurological contouring atlases were developed to uniform this definition and guide clinicians and researchers in assessment of OAR [9], [10], [11].

There is a large interest in developing and validating accurate normal tissue complication probability (NTCP) models to predict a decline in cognitive functioning after radiotherapy. There are however limited high-quality prediction models available. A careful choice of potential predictors and a standardized neurocognitive assessment is necessary [6]. In addition, the availability of a large representative dataset is important to predict and prevent cognitive decline.

To assess short - and long-term neurocognitive decline in patients treated with radiotherapy, and to build a clinical dataset with extensive cognitive follow-up standardized cognitive testing was implemented as a daily clinical routine in our radiotherapy institute for patient cohorts receiving radiotherapy dose to the brain. The aim of this study is to describe the implementation of standardized neurocognitive assessment in clinical routine, by defining the compliance, the method for evaluation of cognitive performance and provide the preliminary results with regard to the number and extend of cognitive changes for patients receiving a radiation dose to the brain.

## Materials and methods

### Patient population

Patients that were treated in the department of Maastricht Radiation Oncology (MAASTRO) as part of their multi-disciplinary oncology treatment. Standardized cognitive assessment is performed since April 2019 for patients with a neurological tumor, patients with head and neck cancer and patients treated with prophylactic cranial irradiation (PCI). Inclusion and exclusion criteria per subgroup were:

Neurological patients: all patients treated for a neurological tumor, including the patients eligible for proton therapy (e.g. glioma, meningioma, chordoma, chondrosarcoma). Excluded are patients with brain metastases or treated with stereotactic radiotherapy.

Patients with head and neck cancer: all patients in whom the brain will receive radiation dose. Excluded are patients who receive re-irradiation, have a skin cancer, early glottic cancer treated with local radiation only and patients treated with palliative intent.

Patients treated with PCI: all patients treated with curative intent (stage I-III small-cell lung cancer; SCLC).

### Cognitive assessment

The cognitive assessments are performed at baseline, 6 months after radiation treatment, 1 year and yearly hereafter (up to 5 years), followed by a long term follow up with an interval of 2.5 years. Patients that did not perform a baseline cognitive assessment are not invited for the follow up assessments. Due to Covid-19 pandemic neuro-cognitive follow-up assessment were temporary cancelled by care-giver. In addition, a time window of ±3 months was applied to allow flexibility in the planning of the neurocognitive tests.

The selection of neurocognitive tests was performed in agreement with the collaboration agreement of the Neuro Dutch Proton Therapy Centers (DUPROTON). Tests were selected based on the International Cognition and Cancer Task Force (ICCTF) and response assessment in neuro-oncology (RANO) recommendations [12], [13], and the potential to serve as an outcome measure to evaluate radiation toxicity [14]. This is in agreement with the recently published EPTN consensus on the follow-up of adult patients with brain tumors treated with irradiation [15]. To minimize burden to patient and resources these 3 cognitive tests were selected and performed within a timeframe of 30 min:

1. The Hopkins verbal learning test revised (HVLT-r): The HVLT-r is a verbal learning and memory test, in which patients need to recall a set of 12 words. The words are learned over the course of three learning trials. The sum of these learning trials, is presented as the total recall (HVLT total). After approximately 20 min a delayed recall trial (HVLT delayed) was completed [16], [17].
2. The trail making test A and B (TMT-A, TMT-B): The TMT is an attention test that measures information processing speed and requires the patient to connect as fast as possible numbers in sequential order (TMT-A) or to connect a combination of numbers and letters in an alternating progressive sequence (TMT-B) with a pencil. The score for each test is the total time in seconds to complete the array, including the time for potential corrections [18], [19].
3. The Controlled oral Word association test (COWA): The COWA is a verbal fluency task in which the patient has to generate words beginning with a specified letter. The test consists of 3 trials of one minute, in each trial a different letter is used. The total score (COWA-total) is the sum of the 3 trials [19], [20].

Multiple versions of the HVLT-r and COWA are available, to avoid a learning effect. The patients receive different versions, e.g. a different set of words or letters during each follow-up assessment. For the TMT-A and TMT-B only one version is available.

In addition to cognitive testing, education level was assessed and graded according to the Dutch Verhage scale (1964) [21].

### Statistical analysis

Compliance was calculated defining the patients participating in cognitive assessment, divided by the number of patients who were eligible and invited for cognitive assessment at that timepoint. Patients with progression were eligible for assessment, patients that deceased during the follow-up period were not. Cancelled appointments by the caregiver (e.g. due to covid-19) are also not part of the compliance assessment. The percentage of reasons for non-compliance was calculated by dividing the number of patients per specific reason by the total of non-compliers.

The Reliable Change Index (RCI) was calculated by dividing the difference between pre-treatment (baseline) and posttreatment (6 months or 1 year) scores by the standard error of the difference. The standard error of the difference was defined based on the standard error of the measurement (SEM) and the reliability of the measure (r) from available test–retest data from literature, for HVLT [17], COWA [20], and TMT [18].

A RCI < -1.5or RCI > 1.5 was defined as a significant decline in cognitive functioning. Note that for HVLT and COWA a decrease in test score represents a decline (total number), while for TMT A and TMT B, an increase in test score represents a decline in neurocognitive functioning (time needed). In addition, patients with a significant decrease or increase on multiple test-variables were quantified. A compound score was calculated by defining for each variable if there was a significant decrease (-1), stable measurement (0) or increase (1). The sum of all these values was used as a total score, providing a scale from −5, significant decline on all variables, to + 5, significant improvement on all variables. Using this method, a significant increase on one test variable, can compensate for a decline on a different variable.. Finally, a linear regression was performed to evaluate the relationship between the reliable change index between baseline and 6 months versus baseline and 1 year for the 5 cognitive test variables. The R-squared and p-value of the model are presented.

## Results

### Implementation and data acquisition

At the time of clinical implementation (April 2019) 75% (9 out of 12) of the radiation oncology nurses were certified by the European Organization for Research and Treatment of Cancer (EORTC) to perform the neurocognitive assessment. After 7 months all radiation oncology nurses were trained and certified. Certification was obtained by 1) completing an educational lecture on neurocognition, 2) an online training program including a test provided by the EORTC, 3) an assessment in which the tests were performed, first on a volunteer and second, on a patient under the supervision of a qualified trial assistant, 4) unsupervised assessment in 5 patients, followed by a 6th supervised assessment. To guarantee the quality and a uniform neurocognitive assessment, the provided certification is assessed every 6 months by a qualified trial assistant.

The neurocognitive test results are reported in the electronic patient record (HIX version 6.1, Chipsoft, Amsterdam, Netherlands). From the patient record, results are queried and a Business Intelligence (BI) report generated, to provide insight into the number of tests performed. In addition, the causes of non-compliance or incomplete results can be monitored, which allows corrections to process and/or data collection in an early stage. Also, it enables the selection of sub-populations of patients for further analysis and allows to evaluate performance over time on an individual level. The patient and/or treating physician can request the results of the test in a written report.

### Patients

In December 2021 a cross-sectional data-extraction was performed. Between April 2019 and December 2021, 1187 neurocognitive tests were performed, 650 patients performed neurocognitive tests at baseline, 336 patients at 6 months and 201 at 1 year after RT. See Table 1 for patient characteristics.

**Table 1:** Patient characteristics from the patients that performed cognitive tests at baseline, 6 months (6 M) and 1 year (1Y) after treatment. The education level according to Verhage (1964) [21]. The subgroups of patients with a neurological tumor (Neuro), head and neck (HN) lesion and patients treated with PCI.

	**Baseline**		**6 M after RT**		**1Y after RT**	
**Patients*(N)***	650		336		201	
**Gender**	*N*	*%*	*N*	*%*	*N*	*%*
Female	266	41	142	42	84	42
Male	384	59	194	58	117	58
**Age (***Mean±SD)*	63 ± 13y (19-93y)		61 ± 13y (19-93y)		60 ± 14y (19-93y)	
**Education level**						
*1. less than 6 years of primary education*	9	1	8	2	2	1
*2. finished primary education*	39	6	13	4	9	4
*3. primary education and less than 2 years of low-level secondary education*	63	10	28	8	16	8
*4. finished low-level secondary education*	145	22	71	21	45	22
*5. finished average level secondary education*	197	30	106	32	57	28
*6. finished high level secondary education*	148	23	81	24	54	27
*7. university degree*	34	5	23	7	15	7
*Other*	1	0	0	0	0	0
*NA*	14	2	6	2	3	1
**Sub-group**						
Neuro	249	38	134	40	74	37
HN	313	48	175	52	106	53
PCI	88	14	27	8	21	10
**Radiation Treatment**						
Neuro 15c2.67 Gy 26x1.8 Gy 28x1.8 Gy 29x1.8 Gy 30x1.8 Gy 30x2Gy 33x1.8 Gy 1x15Gy	- - - - - - - -		9 10 45 1 16 27 25 1	7 7 34 1 12 20 19 1	2 5 29 1 10 7 19 1	3 7 39 1 14 9 26 1
HN						
- 23x2Gy - 28 × 2 Gy - 33 × 2 Gy - 34x2Gy - 35x2Gy	- - - - -		1 7 37 48 82	1 4 21 27 47	1 7 18 27 53	1 7 17 25 50
PCI						
- **10x2.5 Gy**	-		27	100	21	100
**Concurrent chemotherapy**						
Neuro Yes No			34 100	25 75	9 65	12 88
HN Yes No			64 111	37 63	39 67	37 63

From the included patients, 7% (N = 46) deceased within 6 months after start radiotherapy and 20% (N = 131) 1 year after start radiotherapy. For the individual sub-groups the numbers are within 6 months; HN: 10/313, PCI 21/88, Neuro 15/249 and within 1 year; HN: 41/313, PCI 40/88, Neuro 50/249. Note that given the cross-sectional nature of the dataset, for not all patients with a baseline cognitive assessment the 6 month or 12-month follow-up period was completed.

### Compliance

**Fig. 1:**
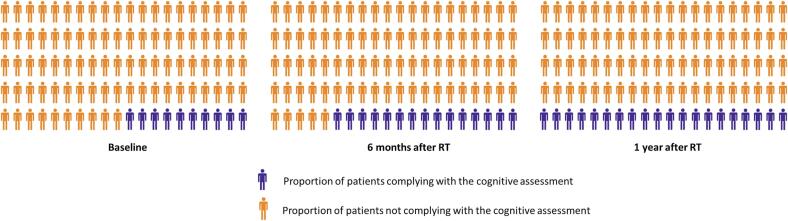
Visualization of the compliance to the neurocognitive assessment at baseline, 6 months and 1 year after radiotherapy.

The compliance to the neurocognitive tests was for the total group 90.4%, 85.6% and 75.3% at baseline, 6 months and 1 year respectively (Fig. 1). For the subgroups the compliance were, 100% (baseline) 75,0% (6 months) and 85% (1 year) for the PCI group. 88.9% (baseline), 85.5% (6 months), 87.8% (1 year) for the HN population and 94.3% (baseline), 86.9% (6 months) and 71.7% (1 year) for the group with a neuro-oncological lesion. The four main reasons for non-compliance were observed: 1. Patient did not attend appointment for unknown reason (49.2%), 2. Patient was unable to perform the test due to illness (12%), 3. Patient refused to perform the test (8%), 4. Other reasons, (31%), including difficulty speaking, hearing problems, previous irradiation and fear of Covid-19.

### Cognitive performance

The average values, standard deviation and reliable change index of the neurocognitive test variables are presented in Table 2 for the total population. In Supplementary Table this is available for the individual sub-groups.

#### 6 months after treatment

At 6 month a decline was observed in 19% of the patients for immediate recall, 17% for delayed recall, 8% for verbal fluency, 12% for processing speed and 17% for cognitive flexibility. An increase in cognitive performance was observed in a proportion of the patients on all test variables, respectively 15%, 13%, 6%, 25% and 25% at 6 months for immediate recall, delayed recall, fluency, speed and flexibility, respectively (Table 1). In total, the largest number of patients, 19% (64/336) showed a decline on immediate recall. For the individual sub-groups this percentage ranged from 16% (28/175) for the patients with head and neck cancer, to 20% (27/134) for the patients with a neurological tumor and 33% (9/27) for the patients which received PCI.

**Table 2:** Average ± SD for the variables on the neurocognitive tests at baseline, 6 months (6 M) and 1 year (1Y) after radiotherapy. The average reliable change index (RCI) for these variables at 6 months (6 M; N = 336) and 1 year (1Y, N = 201), and the percentage of patients with a significant decline or increase on this variable.

	**Baseline**	**6 M**	**1Y**	**RCI − 6 M**	**sig. decline 6 M**	**sig. increase 6 M**	**RCI − 1Y**	**sig. decline 1Y**	**sig. increase 1Y**
Immediate Recall (HVLT)	24 ± 6	24 ± 7	25 ± 6	−0.2 ± 1.7	19% (64/336)	15% (49/336)	−0.0 ± 1.5	19% (38/201)	15% (30/201)
Delayed Recall (HVLT)	8 ± 3	8 ± 3	9 ± 3	−0.1 ± 1.7	17%(56/325)	13%(41/325)	−0.1 ± 1.5	17% (33/196)	11% (21/196)
Phonemic fluency (COWA)	32 ± 12	32 ± 13	36 ± 14	−0.1 ± 1.2	8%(28/334)	6%(21/334)	0.4 ± 1.2	5% (10/200)	16% (32/200)
Processing speed (TMT-A) [s]	50 ± 34	45 ± 46	40 ± 35	−0.8 ± 8.1	12%(40/328)	25%(82/328)	−0.9 ± 5.9	14% (28/199)	33% (65/199)
Cognitive flexibility (TMT-B) [s]	116 ± 78	105 ± 89	100 ± 82	−1.1 ± 6.2	17%(55/322)	25%(80/328)	−0.5 ± 5.1	16% (31/199)	26% (52/199)

**Fig. 2:**
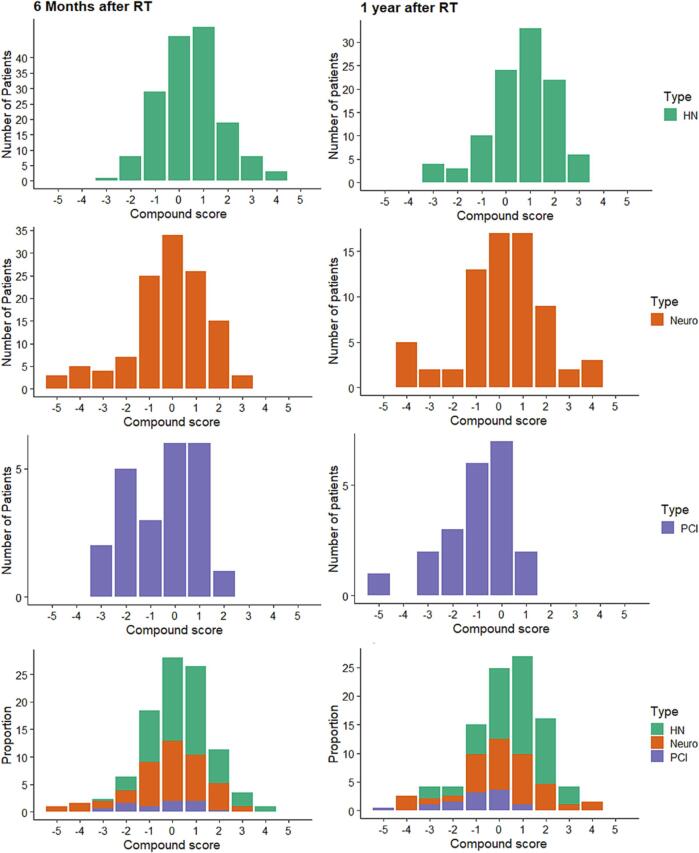
Number of patients with a significant change on the five selected variables per sub-type (head and neck; HN, Neurological (neuro), prophylactic cranial irradiation (PCI) computed in the compound score. Where −5 represents a significant decline on all variables, 5 a significant improvement on all variables. A decline in one variable is compensated for improvement on another variable. The bottom row shows the proportion of patients with a significant decline as a proportion of the total number of patients.

In total, 30% of the patients had a compound score ≤ -1, representing a significant decline on more than one variable at 6 m. 11% of the patients had a significant decline on at least 2 variables. An increase was observed in 42% for one variable (compound score ≥ 1), and 16% for 2 variables (compound score ≥ 2). The distribution of the compound score for the individual subgroups and the total is presented in Fig. 2.

#### 1 year after treatment

At 1y we observed a significant decline in 19% for total recall, 17% for delayed recall, 5% for verbal fluency, 14% for speed and 16% for flexibility. Again, the largest number of patients showed a decline on immediate recall (19% (38/201)). For the individual sub-groups this percentage ranged from 11% (12/106) for the patients with head and neck cancer, to 26% (19/74) for the patients with a neurological tumor and 33% (7/21) for the patients which received PCI.

Also, at 1 year after treatment an increase in cognitive performance was observed in a proportion of the patients, respectively 15%, 11%, 16%, 33% and 26% for immediate recall, delayed recall, fluency, speed and flexibility, respectively (Table 1). The largest group of patients 33% (65/199) improved on speed (TMT-A), over the sub-populations this improvement in speed was observed in 34 % of the patients with head and neck cancer 36% in the neuro group and 14% in the PCI population.

**Fig. 3:**
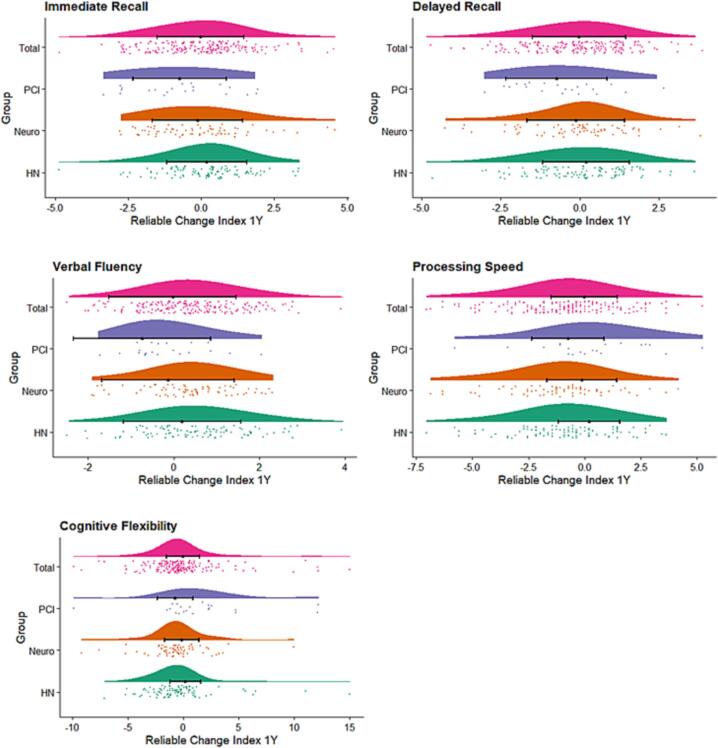
Reliable Change index at 1 year versus baseline, for the 5 variables on the cognitive tests; Immediate recall, delayed recall, verbal fluency, processing speed and cognitive flexibility. Results are shown for the total group (N = 201) and the subgroups of Neuro, Head and Neck (HN) and patients treated with Prophylactic cranial irradaitin (PCI). The black line represents the mean +/- SD.

In Fig. 3 the reliable change index at 1 year after treatment, is visualized for the 5 variables, for the total group of patients and the individual sub-populations.

In total, 26% of the patients showed a significant decline in at least one of the neurocognitive tests at 1y ((compound score ≤ −1), and 11% on at least 2 of these neurocognitive tests at 1y (compound score < −2). An increase was observed in 49% for one variable (compound score ≥ 1), and 22% for 2 variables (compound score ≥ 2). In Fig. 2 the compound score is visualized for the number of patients per sub-group and for the total group.

**Fig. 4:**
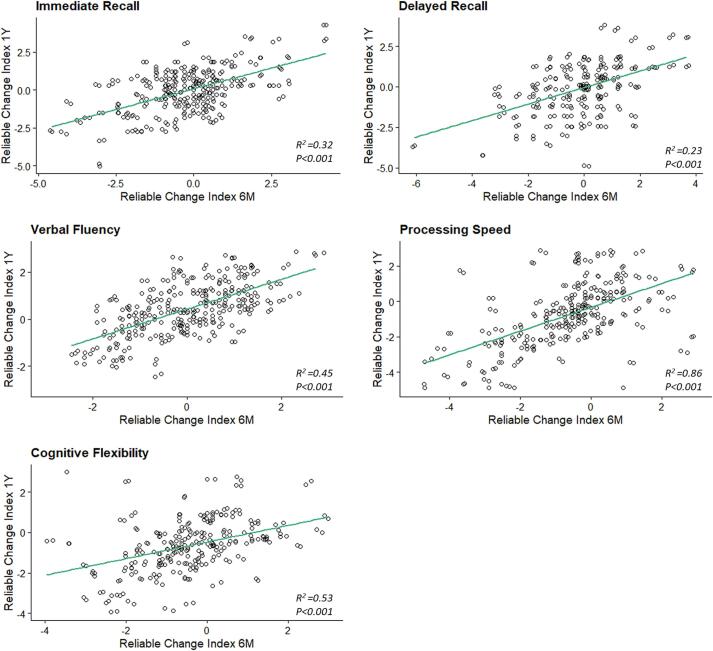
Linear regression between the reliable change index at 6 months and 1 year for the individual test variables. Reported are the R-squared and p-value.

In Fig. 3 the reliable change index is visualized for the 5 variables, for the total group of patient and the individual sub-populations (Fig. 4).

#### 6 months versus 1 year after treatment

The relationship between the reliable change index at 6 months versus 1 year for the 5 variables are presented in Fig. 3. The R-squared were 0.32, 0.23, 0.45, 0.86, and 0.53 for HVLT total, HVLT delayed, COWA, TMT-A and TMT-B respectively, all with a p value <0.001 indicating that the change in cognitive performance at 6 months is related to the change measured at 1 year after treatment.

## Discussion

The aim of this study was to present the clinical implementation of standardized neurocognitive assessment in the radiation oncology clinical routine, provide the compliance, method for evaluation and the preliminary results with regard to the number and extend of cognitive change for patients that received radiation dose to the brain.

In our clinic we experienced that the implementation of neurocognitive assessment as part of clinical routine is feasible. A high compliance rate at baseline and during follow-up was achieved, with >75% compliance after one year. The follow-up compliance is comparable to the compliance previously published by Regine et al. [14] within a multi-institutional trial in patients with brain metastases. In their study the follow-up compliance rate was 78% for HVLT, 80% for COWA and 73% for TMT-A&B. Reasons for non-compliance were similar, including the majority of patients refusing the tests. In our study we observed that the most reported reason was not showing up for the appointment. Note that, in our clinical cohort, patients do not have a regular follow-up with their radiation oncologist, an additional appointment is made cognitive testing in follow up, which might influence compliance. Additional, reasons for differences in observed compliance rate can most probably be explained by the differences in the patient population between both studies and COVID-19.

In this interim analysis the changes in cognition after treatment with radiotherapy were assessed at 6 months and 1 year after treatment to evaluate the current process, methodology and test performance. The proportion of patients that showed a decrease in cognitive performance is in the same range as the proportion of patients that show an increase. The significant decrease that was observed in 26% of the patients at 1 year. This is less than previously reported by Makale et al. [3] who reported 50–90% of patients who were confronted with cognitive dysfunction. An increase in neurocognitive performance was observed in 49% of the patients on more than one of the test variables at 1 year after treatment. An improvement could occur due to tumor regression, for the patients with a lesion in the brain, however also bias due to repeated testing or patient bias can have an impact. In detail, most patient improved in speed and cognitive flexibility, the TMT-A and TMT-B, respectively. For these tests only one version is available, which can cause a repeated test bias in the measurement. In addition, the patients that survive and comply to neurocognitive testing are potentially patients with a good performance. Also, in the current study we based the change in cognitive performance on the objective instantaneous measurements of neurocognition using validated tests. There might be a difference in neurocognitive test results and patient experience, for example obtained with patient reported outcomes or the cognitive failure questionnaire. A recent study of van der Gucht [22] showed for example that the use of mindfulness can provide a positive effect on the subjective measures of cognitive impairment and functional MRI, bus had no effect on the objective neurocognitive assessment. The disability emerging from neurocognitive will therefore always be a combination of measurable effect and perceived impairment by the patient.

This discrepancies in neurocognitive outcomes can make it difficult to define the best endpoint for NTCP models. As presented in the review by Tohidenezhad et al. [6] the currently used predictors and outcomes variables are diverse, nevertheless the HVLT, COWA and TMT were most often used and are part of the EPTN consensus guidelines for follow-up [15]. In addition, the follow-up timepoint ranged from 6 months to 12 years. A straightforward consensus should be made on the outcome assessment and follow-up timepoints to generate a broad applicable NTCP model.

Although implementation of neurocognitive testing in clinical practice was successful with a high compliance, the increasing patient population and follow-up visits can become time and labor intensive. Multiple digital solutions have been developed to assess cognitive abilities. For example, the validity and reliability of the Amsterdam Cognition Scale was investigated in 96 cancer patients, and was shown to be a highly usable tool to measure cognitive abilities [23]. In addition, Mindmore, a comprehensive cognitive screening battery was designed which includes 14 traditional cognitive tests [24]. This was evaluated in comparison with standard neurocognitive assessment in 81 healthy adults and showed to be comparable to the traditional paper-based assessment [25]. The potential of digital solutions for standard neurocognitive assessment might provide significant benefit to facilitate large-scale assessment of cognitive abilities.

This study has several limitations, 1) For the TMT there is only one version available, this could generate a repetition bias, and therefore increased performance over time. 2) Due to COVID-19 pandemic, appointments were for a period delayed or cancelled by the caregiver, these patients were excluded from the eligible population at that timepoint but caused a drop in participant number in follow-up. 3) The follow-up of 1 year is limited, permanent cognitive changes can occur at a later stage, therefore follow-up of this population will be extended and reported at a later stage. 4) All available data was used to evaluate compliance but also generates a very diverse set of patients to describe cognitive performance. Further sub-specifications, analysis and NTCP modelling will be performed as sample size and follow-up increases.

## Conclusions

Standardized neurocognitive testing within the radiotherapy clinic was successfully implemented, with a high patient compliance of 75% at 1 year, independent of patient group. A semi-automatic method to evaluate the cognitive performance after radiation treatment was defined. One year after treatment, patients treated with a radiation dose to the brain showed significant changes in cognitive performance. Data collection is ongoing, long term follow-up (up to 5 years after treatment) and dose–effect analysis will be performed.

## Declaration of Competing Interest

The authors declare that they have no known competing financial interests or personal relationships that could have appeared to influence the work reported in this paper.

## Appendix A Supplementary data

1
