# Supplementary material for: Clinical implementation of standardized neurocognitive assessment before and after radiation to the brain
Source: Clin Transl Radiat Oncol. 2023 Jul 22;42:100664. doi: 10.1016/j.ctro.2023.100664 (PMC10413416; doi:10.1016/j.ctro.2023.100664)
Supplement: 1 [file mmc1.docx]

Supplementary Tables: Average ± SD for the variables on the neurocognitive tests at baseline, 6 months (6M) and 1 year (1Y) after radiotherapy for the individual sub-groups of patients; head and neck (HN), neurological (Neuro) or treated with prophylactic cranial irradiation (PCI). The average reliable change index (RCI) for these variables at 6 months (6M) and 1 year (1Y), and the percentage of patients with a significant decline or increase on this variable.

| **HN** | **Baseline** | **6M** | **1Y** | **RCI - 6M** | **sig. decline 6M** | **sig. increase 6M** | **RCI - 1Y** | **sig. decline 1Y** | **sig. increase 1Y** |
| --- | --- | --- | --- | --- | --- | --- | --- | --- | --- |
| Immediate Recall (HVLT) | 24±6 | 25±6 | 25±6 | 0.0±1.5 | 16% (28/175) | 18% (31/175) | 0.2±1.4 | 11% (12/106) | 17% (18/106) |
| Delayed Recall (HVLT) | 8±3 | 8±3 | 9±3 | 0.0±1.5 | 15% (26/171) | 15% (26/171) | 0.0±1.4 | 13% (14/104) | 12% (12/104) |
| Phonemic fluency (COWA) | 32±12 | 34±13 | 37±13 | 0.1±1.1 | 5% (8/174) | 9% (15/174) | 0.5±1.2 | 4% (4/105) | 20% (21/105) |
| Processing speed (TMT-A) [s] | 46±24 | 38±17 | 37±16 | -0.8±2.3 | 7% (12/172) | 27% (46/172) | -0.8±2.1 | 11% (12/105) | 34% (36/105) |
| Cognitive flexibility (TMT-B) [s] | 114±70 | 94±62 | 93±65 | -0.7±3.1 | 11% (19/171) | 30% (51/171) | -0.5±2.9 | 9% (9/105) | 29% (30/105) |

| **NEURO** | **Baseline** | **6M** | **1Y** | **RCI - 6M** | **sig. decline 6M** | **sig. increase 6M** | **RCI - 1Y** | **sig. decline 1Y** | **sig. increase 1Y** |
| --- | --- | --- | --- | --- | --- | --- | --- | --- | --- |
| Immediate Recall (HVLT) | 24±7 | 24±8 | 25±7 | -0.3±1.8 | 20% (27/134) | 11% (15/134) | -0.1±1.5 | 26% (19/74) | 12% (9/74) |
| Delayed Recall (HVLT) | 8±3 | 8±4 | 9±3 | -0.3±2.0 | 21% (27/129) | 10% (13/129) | -0.1±1.5 | 17% (12/71) | 11% (8/71) |
| Phonemic fluency (COWA) | 32±13 | 32±14 | 37±16 | -0.2±1.3 | 13% (17/133) | 5% (6/133) | 0.5±1.3 | 7% (5/74) | 14% (10/74) |
| Processing speed (TMT-A) [s] | 54±41 | 54±70 | 43±53 | 1.7±10 | 19% (25/129) | 24% (31/129) | 0.0±8.3 | 14% (10/73) | 36% (26/73) |
| Cognitive flexibility (TMT-B) [s] | 122±86 | 115±117 | 100±103 | 0.8±6.5 | 21% (27/126) | 21% (26/126) | 0.1±5.5 | 18% (13/73) | 29% (21/73) |

| **PCI** | **Baseline** | **6M** | **1Y** | **RCI - 6M** | **sig. decline 6M** | **sig. increase 6M** | **RCI - 1Y** | **sig. decline 1Y** | **sig. increase 1Y** |
| --- | --- | --- | --- | --- | --- | --- | --- | --- | --- |
| Immediate Recall (HVLT) | 25±5 | 22±6 | 22±5 | -0.5±1.7 | 33% (9/27) | 11% (3/27) | -0.7±1.6 | 33% (7/21) | 14% (3/21) |
| Delayed Recall (HVLT) | 8±3 | 8±3 | 8±2 | -0.2±1.2 | 12% (3/25) | 8% (2/25) | -0.6±1.4 | 33% (7/21) | 5% (1/21) |
| Phonemic fluency (COWA) | 31±11 | 27±11 | 30±10 | -0.5±1.2 | 11% (3/27) | 0% (0/27) | -0.2±0.9 | 5% (1/21) | 5% (1/21) |
| Processing speed (TMT-A) [s] | 52±40 | 46±20 | 47±19 | -0.19±2.4 | 11% (3/27) | 19% (5/27) | 0.3±2.4 | 29% (6/21) | 14% (3/21) |
| Cognitive flexibility (TMT-B) [s] | 112±78 | 127±67 | 124±73 | 1.6±4.6 | 36% (9/25) | 12% (3/25) | 1.5±4.6 | 43% (9/21) | 5% (1/21) |
